# Converting non-metallic printed circuit boards waste into a value added product

**DOI:** 10.1186/2052-336X-11-2

**Published:** 2013-05-10

**Authors:** Shantha Kumari Muniyandi, Johan Sohaili, Azman Hassan, Siti Suhaila Mohamad

**Affiliations:** 1 Department of Environmental, Faculty of Civil Engineering, Universiti Teknologi Malaysia, 81310 UTM, Skudai, Johor, Malaysia; 2 Department of Polymer Engineering, Faculty of Chemical Engineering, Universiti Teknologi Malaysia, 81310 UTM, Skudai, Johor, Malaysia

**Keywords:** Nonmetallic, Printed circuit board, Mechanical properties, Morphological, Compatibilizer

## Abstract

The aim of this study was to investigate the feasibility of using nonmetallic printed circuit board (PCB) waste as filler in recycled HDPE (rHDPE) in production of rHDPE/PCB composites. Maleic anhydride modified linear low-density polyethylene (MAPE) was used as compatibilizer. In particular, the effects of nonmetallic PCB and MAPE on mechanical properties of the composites were assessed through tensile, flexural and impact testing. Scanning electron microscope (SEM) was used to study the dispersion of nonmetallic PCB and MAPE in the matrix. Nonmetallic PCB was blended with rHDPE from 0–30 wt% and prepared by counter-rotating twin screw extruder followed by molding into test samples via hot press for analysis. A good balance between stiffness, strength and toughness was achieved for the system containing 30 wt% PCB. Thus, this system was chosen in order to investigate the effect of the compatibilizer on the mechanical properties of the composites. The results indicate that MAPE as a compatiblizer can effectively promote the interfacial adhesion between nonmetallic PCB and rHDPE. The addition of 6 phr MAPE increased the flexural strength, tensile strength and impact strength by 71%, 98% and 44% respectively compared to the uncompatibilized composites.

## Introduction

In recent years throughout the world including Malaysia, there has been increasing concern about the growing volume of end of life electronics and the fact that much of it is consigned to landfill without any attempt being made to recycle the nonmetallic materials it contains. A large amount of nonmetallic materials in PCBs are disposed of by combustion and disposal in landfill as the main method for treating nonmetals in PCBs, but it may cause secondary pollution and resource wasting. The problem is generally focused on the non metallic materials since it is being noted by Department of Environment (DOE) Malaysia as hazardous and being listed under SW 501/ SW 110 of the Environmental Quality (Scheduled Waste) Regulations 2005. Since it contains chemical hazards [1], hence it needs to be disposed at licensed scheduled waste disposal site which is Kualiti Alam Sdn. Bhd. The problems arise as the cost of disposal of these hazardous residues is so expensive. Kualiti Alam Sdn. Bhd charge RM150 / tonne of PCB together with other charges including cost of packaging, segregation, transportation and others. Instead of that, these residues are capable to give risk to the human health and surrounding environment if it is not being properly managed [1]. Once PCBs are being filled, it will poses significant contamination problems at which the landfills will leach the toxins into the groundwater [2]. Recently many studies have been carried out to reuse these nonmetallic PCBs in a more profitable and environmentally friendly way. Guo et al., used nonmetals in production of Wood Plastic Composites (WPC), and they found out that the flexural strength of the composites were slightly greater than those of control specimens [3]. Wang and his co workers [4] reported that using of nonmetallic powder from PCBs as an additive in PVC substrate, increased the bending strength over pure PVC with incorporation of 20 wt% PCB. Franz [5] reported that the use of the nonmetallic PCB for thermoplastics would be the perfect recycling solution. To the best of our knowledge, no study on recycled HDPE (rHDPE) and nonmetallic PCB composites modified with MAPE compatibilizer has been reported yet.

High density polyethylene (HDPE) is the thermoplastics widely used as packaging (bottles, films, etc.) and contributes about 72% of total plastics used in rigid containers [6]. This plastic is chosen to study the blend properties since it is a major portion of the post-consumer household wastes. However, the blends of rHDPE/PCB composites are in immiscible due to weak interfacial adhesion between these two phase system which will affect the physical and mechanical properties of the blends. Compatibilizer is generally needed to improve the adhesion and enhance the properties of the polymer blends [7].

In this article, the objective of the research is to study the feasibility of reusing nonmetallic PCBs recycled from waste PCBs as filler material in the rHDPE composites. The effects of nonmetallic PCB and MAPE contents on mechanical and morphological properties of rHDPE/PCB composites were also determined. The rHDPE composites with maximum content of the nonmetallic PCB in the experiment (30 wt%) were selected and tested the leaching characteristics, with the aim to recycled the waste PCBs in a more environmentally sound way.

## Materials and methods

**Figure 1:**
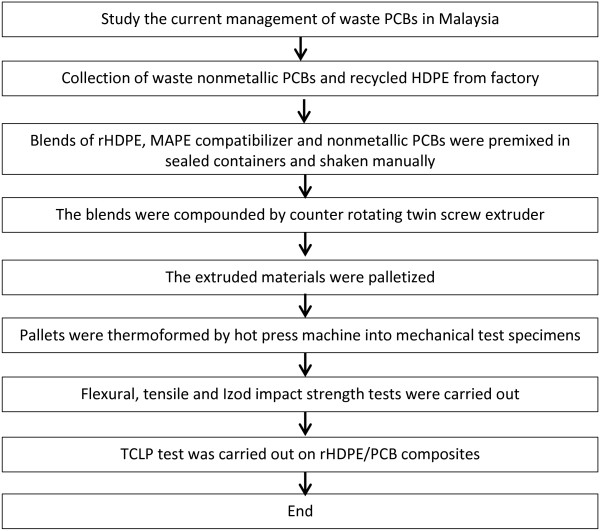
**Research methodology flow chart.** The flow chart shows the steps in production of rHDPE/PCB composites including the testing carried out.

A flow chart is done to arrange and explain all the main activities that have been carried out throughout the research including the steps in production of rHDPE/PCB composites and all the testing. Figure 1 shows the flow chart of research methodology.

### Materials

Table 1 and 2 show the polymer materials, nonmetallic PCBs and the blends system used in this study, respectively. The recycled HDPE (rHDPE) was supplied by a local recycling company in Johor, METAHUB Industries Sdn Bhd. The Maleic anhydride modified linear low-density polyethylene (MAPE) compatibilizer used was OREVAC® 18365 supplied by Arkema. The nonmetallic PCB was an industrial solid-waste byproduct, from METAHUB Industries Sdn Bhd (Johor, Malaysia). This was in the form of powder.

**Table 1:** Designations of rHDPE/PCB and their compositions

**Designation**	**Composition**	**wt %**	
		**rHDPE**	**PCB**
rHDPE	rHDPE	100	-
H90/P10	rHDPE / PCB	90	10
H80/P20	rHDPE / PCB	80	20
H70/P30	rHDPE / PCB	70	30

**Table 2:** Designations of rHDPE/PCB (70/30) with different content of MAPE

**Designation**	**Composition**	**wt %**		**phr**
		**rHDPE**	**PCB**	**MAPE**
H70/P30	rHDPE /PCB/MAPE	70	30	-
H70/P30/M3	rHDPE /PCB/MAPE	70	30	3
H70/P30/M6	rHDPE /PCB/MAPE	70	30	6
H70/P30/M12	rHDPE /PCB/MAPE	70	30	12
H70/P30/M18	rHDPE /PCB/MAPE	70	30	18

### Preparation of nonmetallic PCBs

**Figure 2:**
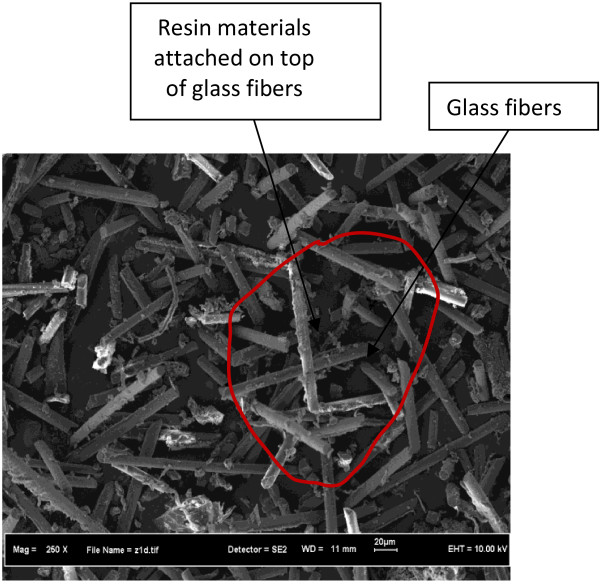
**SEM micrograph of nonmetallic materials from waste PCBs.** Microscopic observation shows that most of the nonmetallic PCBs are single glass fibers and thermosetting resin powders.

The waste nonmetallic PCBs used in this study are without electronic elements. A stack of five sieves with hole widths from 0.3 to 0.07 mm were selected. The PCBs were sieved to remove impurities and manually sieved according to BS 812 sieve test: Part 103: Section 1 [BSI, 1989]. The specimens were agitated for 20 minutes and the nonmetallic PCBs collected on each sieve were weighed to calculate the particle size distribution. The size distribution of nonmetallic PCB materials is shown in Table 3. The nonmetallic PCBs with particle size of less than 0.3 mm were selected for making composites. Microscopic observation shows that most of them are single glass fibers and thermosetting resin powders (Figure 2).

**Table 3:** Size distribution of nonmetallic PCBs materials

**Sieve size (mm)**	**Cumulative percent retained (%)**
0.3-0.15	50
0.15-0.9	25
0.09-0.07	14.3
<0.07	10.7

A series of Energy – dispersive X-ray spectroscopy (EDS) analysis have been carried out to determine the chemical composition of these nonmetallic PCBs. It was found that the nonmetallic PCBs contained approximately 5% of metallic materials such as Cu, Ni, As, and Sn, approximately 60% organic resin materials containing elements such as C and H, and approximately 33% glass fibers materials such as SiO2, MgO,Al2O3. These glass fibers possess many excellent characteristics, such as high length diameter ratio (L/D ratio), high elastic modulus, low elongation and low thermal conductivity [8].

### Compounding and preparation of composites

Blends of rHDPE, MAPE compatibilizer and nonmetallic PCBs, were premixed in sealed containers and shaken manually. The rHDPE and the nonmetallic PCBs were dried at 80°C for 24 hours prior to compounding. The nonmetallic PCBs were added to the rHDPE substrate at levels of 10%, 20% and 30 wt%. Adding larger amount of nonmetallic PCBs (>30 wt%) to rHDPE worsened the processibility of the composite material, and created great difficulty for molding process.

The blends were compounded by simultaneous adding of all components to Brabender Plasticoder PL 2000 counter-rotating twin-screw extruder. The barrel temperature profile adopted during compounding of all blends was 210°C at the feed section, decreasing to 200°C at the die head. The screw rotation speed was fixed at 50 rpm. The selected processing conditions were the optimum conditions. The extruded materials were thermoformed by hot press machine into sheet with thickness of 1mm for tensile specimens and 3 mm thickness for both flexural and impact test specimens according to the specifications that are required in testing sample. The operating temperature was 200°C with 15 minutes of preheat and another 10 minutes for compression, followed by cooling process at room temperature for 5 minutes before removing it from the mold. Preheat was needed to melt the extruded materials and to promote flow of resin to every hill and valley of the fiber. In consequent, there was no significant resin reached or voids introduced. Last but not least, the thin sheets of composites were machined into shape using grinding machine according to standards.

### Measurement of properties

Tensile, flexural and Notched Izod Impact strength tests were carried out according to ASTM D638, ASTM D790 and ASTM 256, respectively, by using an Instron (Bucks, UK) 5567 universal testing machine under ambient conditions. Crosshead speeds of 50 and 3 mm min-1 were used for tensile and flexural testing, respectively. Five specimens of each formulation were tested and the average values reported. The morphology of the composites was examined by using a field emission scanning microscopy, FESEM to analyze the dispersion of fillers into the rHDPE matrix using fractured surfaces. Prior to the analysis, the fractured surfaces of the specimens were sputter coated with a thin layer of gold.

### Preparations of samples for toxicity characteristic leaching procedure (TCLP) test

To conduct this test, the rHDPE/PCB composites in pallet form were taken. Then prepare as much as 2 liters of extraction liquid following the EPA standard TCLP method _EPA 1992a_. Extraction liquid was placed in a bottle High Density Polyethylene (HDPE). Then, a total of 100 gram rHDPE/PCB pallet was placed in HDPE bottles. Sample was prepared with mixing ratio of extraction liquid to composites was 20:1. Bottle is then put into the National Bureau Standards leaching machine. Spin the bottle for 18 hours with a rotation rate of (30 + 2) rpm.

After completion of the leaching process, the sample was filtered with borosilicate glass fiber filter size 0.6 to 0.8 micrometers with 50 psi pressure. This process must be done immediately after the sample was collected. Finally, samples were analyzed by using the ICP MS.

## Results and discussion

### Mechanical properties

#### The effect of non metallic PCB contents

**Figure 3:**
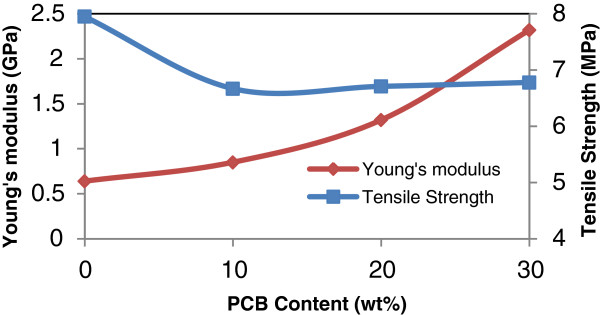
**Effect of PCB contents on the tensile strength and Young’s modulus of rHDPE/PCB composites.** Increases in tensile strength and Young’s modulus were found with additional nonmetallic PCB contents from 10–30 wt%.

**Figure 4:**
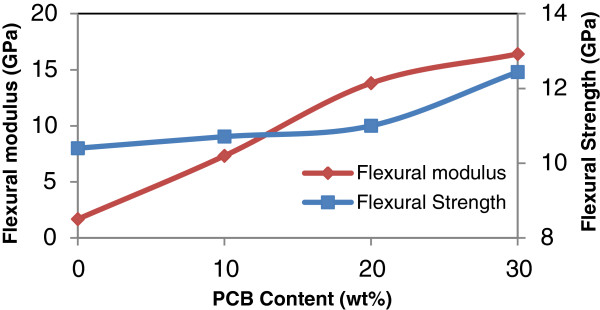
**Effect of PCB contents on the flexural strength and flexural modulus of rHDPE/PCB composites.** Flexural strength and flexural modulus increased with increasing addition of nonmetallic PCB contents from 0–30 wt%.

The effects of various addition of non metallic PCB content (0–30 wt%) on mechanical properties of the rHDPE/PCB composites are given in Table 4. Figures 2, 3 and 4 show the effects of nonmetallic PCB content on the tensile properties of the rHDPE/PCB composites. Interestingly, no significant changes were observed in the tensile strength of rHDPE/PCB composites (Figure 3) with addition of nonmetallic PCBs (0–30 wt%). The factor that possibly contributed to the low tensile strength reported is the less effective adhesion of rHDPE matrix towards nonmetallic PCB particles. The addition of nonmetallic PCB also attributed to the addition of many glass fibers in the composites and this increase the probability of filler agglomeration that create regions of stress concentrations. This agreement with the results presented by Guo et al. whereby they reported that the increasing amount of glass fibers decreased the flow ability of composite and reduced the dispersion of ingredients, which may lead too poor interfacial adhesion [3]. Slight increase in tensile strength without significant changes also revealed that little reinforcement obtained by the addition of filler and possible agglomeration, which weaken the stress transfer from matrix to filler. Another recent study done by Liang and his co-workers found that the molecular chain of polyolefin (PP,PE etc.) is lack of active groups, so it is hardly to form effective interface adhesion because of its poor affinity composite with glass fibers, as a result, stress cannot be transferred to glass fibers well by matrix resin [9]. The enhancement of glass fiber weakens greatly, and this will also influence the performances of the composites.

**Table 4:** Mechanical properties of the rHDPE with various PCB contents

**PCB Content (wt %)**				
Property	0	10	20	30
*Tensile testing*				
Young’s modulus (GPa)	0.64	0.85	1.32	2.32
Tensile strength (MPa)	7.95	6.67	6.71	6.78
Elongation at break (%)	3.5	2.8	2.0	1.6
*Flexural testing*				
Flexural modulus (GPa)	1.68	7.32	13.81	16.41
Flexural strength (MPa)	10.40	10.71	11.00	12.44
*Impact strength* (J/m)	59.6	48.3	44.2	42.5

Young’s modulus of the rHDPE/PCB composites as can be seen in Figure 3, increased steadily as the nonmetallic PCB content increased from (0–30 wt%). The maximum increment of Young’s modulus is 262.5% upon the incorporation of 30 wt% nonmetallic PCB. The increase in the modulus is mainly influenced by the incorporation of rigid fiber/ filler reinforcements into the polymer making it stiffer.

Meanwhile, flexural properties of the rHDPE/PCB composites as can be seen in Figure 4 are improved significantly by adding nonmetallic PCBs (0–30 wt%). Moreover, the increases are greater than that from tensile properties. This to the fact that, glass fibers in nonmetallic PCBs reinforced the properties of composites with mass excellent supporting bodies and appropriate interfacial adhesives formed between the particles and matrix [8]. Flexural strength increased by 4% with the incorporation of 20 wt% nonmetallic PCB and further improvement occurred with further increases in nonmetallic PCB content. When the content of fillers increases to 30 wt%, the maximum increment of flexural strength and modulus of the composites is 20% and 96.7%, respectively. Mou and his co-workers used different proportions of nonmetallic PCB in their studies and compared it with the two typical materials used for making composite boards which are talc and silica powder. Their research showed that the outstanding characteristic of the nonmetallic material board is its flexural strength, which was enchanced by more than 50% for the 15% blending ratio when compared with talc [10]. Therefore, they concluded that this characteristic is good for products that mainly bear bending stresses.

**Figure 5:**
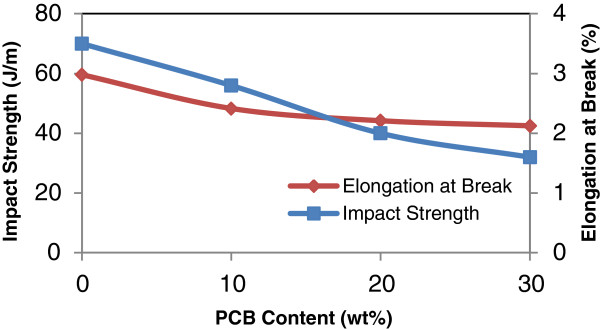
**Effect of PCB contents on the impact strength and elongation at break of rHDPE/PCB composites.** There is a decrease in both the impact strength and the elongation at break with incorporation of 10–30 wt% nonmetallic PCBs contents.

Decreasing in impact strength and elongation at break (Figure 5) with incorporation of 0–30 wt% PCB implies that addition of stiff reinforcements such as glass fibers can reduce the mobility of polymer chain thus restricted its movement [11]. This also can lower the impact strength of the matrix since the reinforcements will cause stress concentrations and exhibited brittle nature. Reduction of extensible matrix in the composites with the increasing content of fillers also can be related to the decreasing in elongation at break. Incorporation of nonmetallic PCB also reduced the compatibility between nonmetallic PCB and rHDPE matrix, which is reflected in the slight decreased in elongation at break and impact strength. Several researchers have studied the trend of increasing tensile modulus and decreasing elongation at break and impact strength with the increasing amount of glass fibers in the composites [12-14]. Studies by Young and Baird on tensile strength, modulus and elongation of phosphate glass in poly (ether ether ketone) (PEEK) and poly (ether imide) (PEI) also showed a similar behavior [15]. These results explained that glass fibers in nonmetallic PCB enhanced the stiffness of the composites but reduced the performance of the toughness.

### The effect of compatibilizer content

From the previous section, it is reasonable to suggest that a good balance between stiffness and strength was achieved for the system containing 30 wt% PCB. Thus, this system was chosen in order to investigate the effect of the compatibilizer on the mechanical properties of the composites. The effects of the composites (MAPE) content on the mechanical properties are depicted in Table 5.

**Table 5:** Mechanical properties of rHDPE/ PCB (70/30) with various MAPE contents

**MAPE (phr)**					
Property	0	3	6	12	18
*Tensile testing*					
Young’s modulus (GPa)	2.32	2.8	3.2	2.34	2.32
Tensile strength (MPa)	6.67	11.35	13.23	12.78	10.25
Elongation at break (%)	1.6	1.9	2.0	2.24	2.22
*Flexural testing*					
Flexural modulus (GPa)	16.41	24.52	27.68	21.61	20.63
Flexural strength (MPa)	12.44	17.50	21.26	20.00	14.26
*Impact strength* (J/m)	42.5	48.4	52.6	61.1	60.0

**Figure 6:**
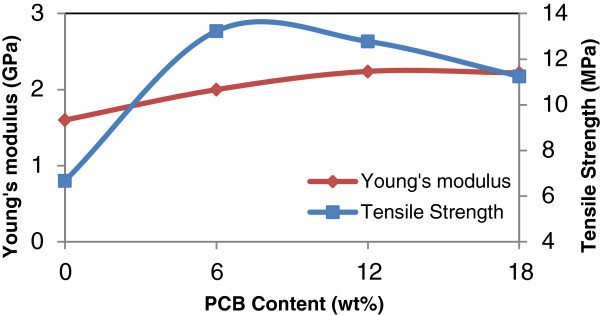
**Effect of MAPE contents on the tensile strength and Young’s modulus of rHDPE/PCB composites.** Tensile strength and Young’s modulus achieved a maximum level when the amount of MAPE compatibilizer was about 6 phr, and further addition of MAPE up to 18 phr caused a slight reduction.

As can be seen in Figure 6, the maximum increment of tensile strength and Young’s modulus is 98% and 38%, respectively with incorporation of 6 phr MAPE. Further addition of MAPE up to 18 phr reported a slight decrease in both tensile strength and modulus.

**Figure 7:**
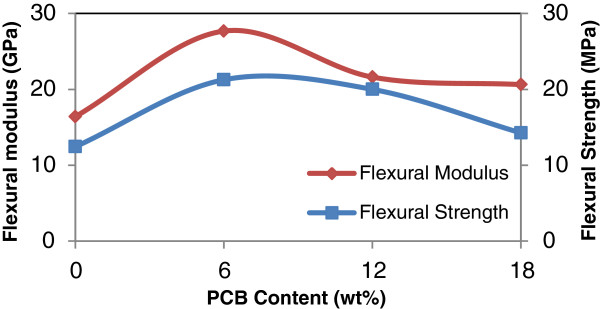
**Effect of MAPE content on the flexural strength and flexural modulus of rHDPE/PCB composites.** Flexural strength and flexural modulus of the composites increased up to 6 phr MAPE content with a decrease with further addition of MAPE.

The same trend was observed in flexural properties (Figure 7). The addition of 6 phr MAPE increases the flexural strength and flexural modulus of composites by 71% and 69% respectively, when compared to uncompatibilized composites. The flexural properties reach maximum at a MAPE content of 6 phr, with a decrease with further addition of MAPE. The improvements in flexural and tensile properties are believed to be due to the MAPE being able to associate with the functionality of the nonmetallic PCB, which enhanced the interfacial adhesion between the nonmetallic PCB and rHDPE matrix.

The presence of excessive compatiblizer amounts causes a significant reduction in tensile and flexural properties of the composites and this demonstrates that excessive MAPE does not favor the improvement of mechanical performances. This is in agreement with Sathe et.al., whereby they stated that up to a saturation level of the compatibilizer, its molecules are located in the interphase between the matrix and the dispersed phase [16]. However, when the concentration of a compatibilizer is above the saturation level, only a part of the molecules locates in the interfacial area, and the excess is dispersed in the matrix affecting its homogeneity and consequently the mechanical properties of the blends.

**Figure 8:**
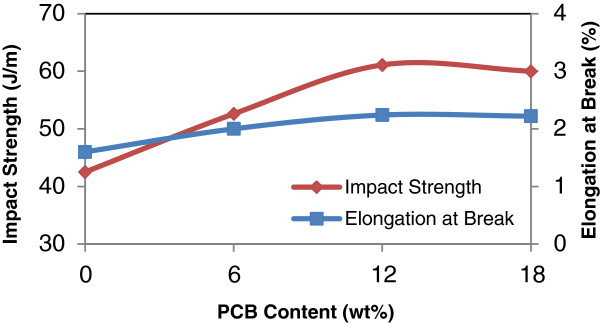
**Effect of MAPE content on the impact strength and elongation at break of rHDPE/PCB composites.** Impact strength and elongation at break increased with the increasing MAPE amount up to 12 phr. Beyond 12 phr, the elongation at break began to achieve constant value.

Same trend were observed in Figure 8 for the impact strength and elongation of break for rHDPE/PCB/MAPE composites whereby for the uncompatibilized rHDPE / PCB composites as expected had a lower value of elongation at break and impact strength. When compatibilizer MAPE was introduced, the rHDPE / PCB system showed an increasing in elongation and impact properties and had caused a balance in properties between the matrix and filler, but beyond 12 phr of MAPE contents, the elongation at break and impact strength began to achieve the constant value. This might happened because at high maleic anhydride contents, incompatibility ‘problems’ could arise. Therefore, high contents of such oligomers can adversely affect the elongation at break and impact properties. It seems that the incorporation of 6 phr MAPE improve on the toughness of the blends with balanced strength and stiffness.

### Field emission scanning electron microscopy (FESEM)

**Figure 9:**
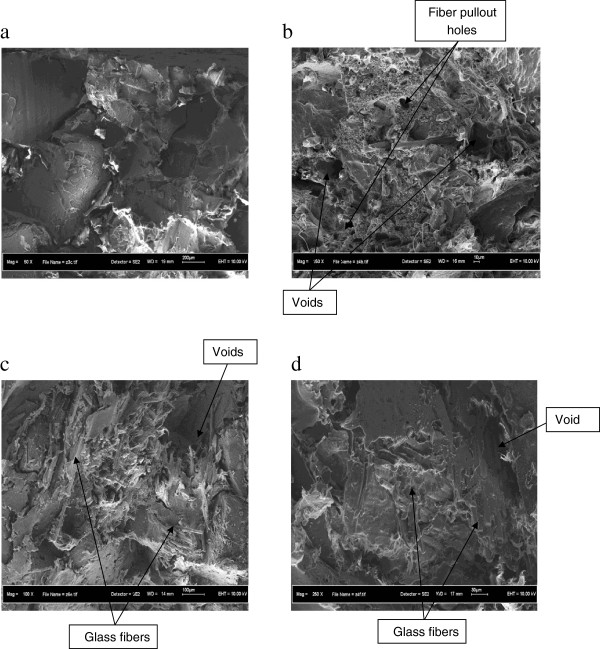
**SEM photographs of the fracture surfaces of rHDPE/PCB specimens with different contents of nonmetallic PCBs: (a) 0 wt%, (b) 10 wt%, (c) 20 wt% and (d) 30 wt%.** Figure 9 a-d show the SEM images of fractured surface of rHDPE/PCB specimens. Without compatibilizer, there were obvious separation between rHDPE and the nonmetallic PCB because of the incompatibility between matrix and fillers.

FESEM was used to examine the morphology of the blends. Figures 9a-d show the SEM images of fractured surface of rHDPE / PCB composites filled with 0 – 30 wt% nonmetallic PCB, respectively.

When the adding content of nonmetallic PCB was 10 wt% and 20 wt%, the composites showed low performances. It can be attributed to a lack of sufficient particle bonding as shown in Figures 9b-c. Deep voids appeared in the matrix of rHDPE/PCB, showing poor adhesion to the matrix.

When nonmetallic PCB materials content was 30 wt%, as can be seen in Figure 9d, glass fibers in nonmetallic PCB adhered onto or inserted into the matrix well compared to 10% and 20 wt% nonmetallic PCBs contents (Figures 9b-c), but there are still some voids can be seen indicating lack of sufficient bonding between particles.

**Figure 10:**
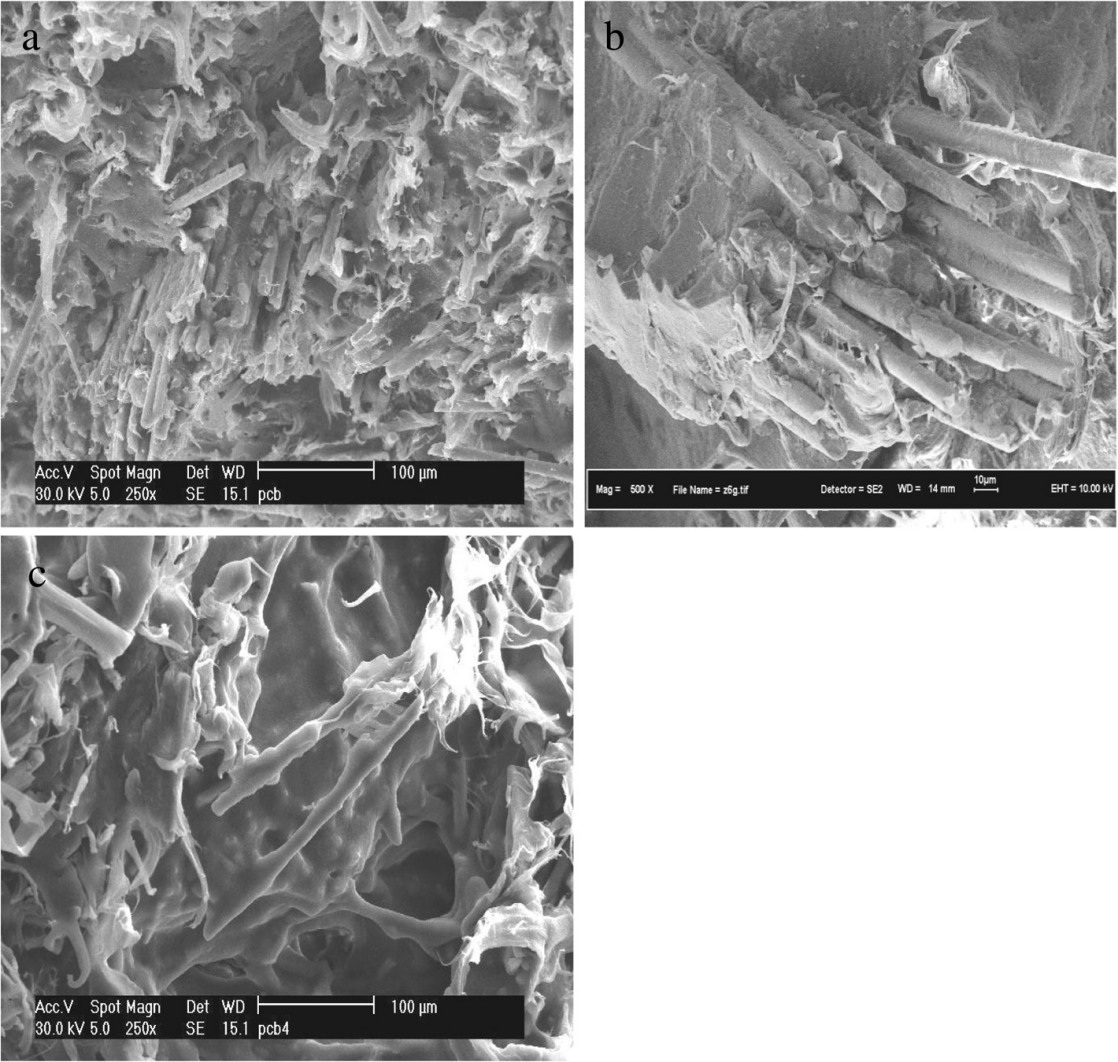
**SEM photographs of the fracture surfaces of rHDPE/PCB/MAPE specimens with different contents of MAPE: (a-b) 6 phr and (c) 18 phr.** The gaps between the matrix and the fillers were reduced when 6 phr of MAPE compatibilizer were added. Less glass fibers pullout and fewer voids were also observed within the matrix indicating MAPE compatibilizer improved the compatibility between the matrix and nonmetallic PCB fillers.

It can be observed that, without compatibilizer, there were obvious separation between rHDPE and the nonmetallic PCB, as shown in Figures 9b-d, because of the incompatibility between matrix and fillers. The gaps between the matrix and the fillers were reduced when 6 phr of MAPE compatibilizer were added (Figure 10a). The adhesion between glass fibers and matrix was seen by a closer observation at higher magnification in Figure 10b. There was filler/matrix filled in the gap of glass fibers, which showed a very strong interfacial bonding between glass fibers in nonmetallic PCBs and recycled HDPE. The good adhesion between glass fibers and matrix can strengthen the mechanical properties of the composites.

The MAPE significantly improved the compatibility between the matrix and nonmetallic PCB fillers, and the matrix was bonded to the fillers well, thus improved the compatibility and interfacial adhesion of the rHDPE/PCB composites. Furthermore, the particles are dispersed well in the matrix and the nonmetallic PCB materials are well wetted with rHDPE material because of good compatibility between nonmetals and matrix. With presence of MAPE compatibilizer, there were no obvious nonmetallic PCB agglomerates within the recycled matrix.

On the other hand, less glass fibers pullout and fewer holes were observed within the matrix in the rHDPE/PCB composites (Figure 10a), and there were also no obvious gaps at the irregular interfaces between the polymer and the glass fibers. With incorporation of 18 phr of MAPE, it can be seen that the excessive use of compatibilizer results in the formation of a weak elastomeric phase that starts to deteriorate the composite’s mechanical properties (Figure 10c) which in agreement to the results obtained in mechanical properties earlier.

### The concentration of heavy metals leached from the rHDPE/PCB composites

TCLP test was carried out to evaluate and determine the concentrations of heavy metals leached from the rHDPE/PCB composites. The EPA standard TCLP method _EPA 1992a_ was employed in this test. The composites with maximum content of the nonmetallic PCBs (30 wt%) were selected and tested the leach concentrations of heavy metals. TCLP tests were done in three replicates and the average value was reported. Table 6 lists the concentration of metals leached from the nonmetallic PCBs and compared with existing standard, which is the Maximum Concentration of Contaminants for the Toxicity Characteristic Leaching Procedure (TCLP) specified under the guideline of DOE. It shows that all the parameters are complied with the standard. Therefore, we can conclude that the nonmetallic PCB is safe to be reuse in rHDPE composites.

**Table 6:** TCLP Result of rHDPE/PCB Composites

**Method reference**	**Parameters**	**Units**	**Results**	**DOE limit**	**Remarks**
USEPA 6010 B	Arsenic	mg/l	0.005	5	Pass
USEPA 6010 B	Barium	mg/l	2.462	100	Pass
USEPA 6010 B	Cadmium	mg/l	0.001	1.0	Pass
USEPA 6010 B	Chromium	mg/l	0.034	5.0	Pass
USEPA 6010 B	Lead	mg/l	0.074	5.0	Pass
USEPA 7470 A	Mercury	mg/l	0.001	0.2	Pass
USEPA 6010 B	Nickel	mg/l	0.046	100	Pass
USEPA 6010 B	Selenium	mg/l	<0.004	1.0	Pass
USEPA 6010 B	Silver	mg/l	<0.001	5.0	Pass
USEPA 6010 B	Tin	mg/l	<0.01	100	Pass
USEPA 6010 B	Zinc	mg/l	0.436	100	Pass
USEPA 6010 B	Copper	mg/l	3.07	100	Pass
USEPA 6010 B	Boron	mg/l	0.12	400	Pass

## Conclusions

The effects of nonmetallic PCBs and MAPE compatibilizer loadings on the morphology and mechanical properties of rHDPE/PCB blends have been investigated. From tensile and flexural tests, the optimum loading of nonmetallic PCB is found to be 30 wt%. Tensile strength, Young’s modulus, flexural strength and flexural modulus increased steadily until reaching maximum at PCB content of 30 wt%. Therefore, the system containing 30 wt% PCB was chosen in order to investigate the effect of MAPE compatibilizer on the mechanical properties of the composites. The addition of 6 phr MAPE increased the flexural strength and flexural modulus of composites by 71% and 69% respectively when compared to uncompatibilized composites. Same trend was observed in tensile properties, whereby tensile strength and Young’s modulus increased with addition of MAPE up to 6phr by 98% and 38% respectively. Further excessive use of MAPE from 12 to 18 phr caused a slight reduction in both flexural and tensile properties. With incorporation of 12 phr MAPE compatibilizer, impact strength and tensile elongation at break increased by 44% and 40% respectively compared to uncompatibilized composites. Beyond 12 phr of MAPE, both impact strength and tensile elongation at break began to achieve constant value. SEM analysis showed that compatibilizer significantly improved the compatibility between the rHDPE matrix and nonmetallic PCB fillers thus, improved the mechanical performances of the composites. So, it can be recommended that a balance in strength, stiffness and toughness of the composites was achieved with the incorporation of 30 wt% PCB and 6 phr MAPE compatibilizer.

## Competing interests

The authors declare that they have no competing interests.

## Authors’ contributions

All the authors have played their roles throughout the completion of this research. All authors read and approved the final manuscript.
